# Core Collections: Is There Any Value for Cotton Breeding?

**DOI:** 10.3389/fpls.2022.895155

**Published:** 2022-04-28

**Authors:** Lucy Marie Egan, Warren Charles Conaty, Warwick Nigel Stiller

**Affiliations:** CSIRO Agriculture and Food, Narrabri, NSW, Australia

**Keywords:** core collection, germplasm management, cotton, *Gossypium*, breeding

## Abstract

Global plant breeding activities are reliant on the available genetic variation held in extant varieties and germplasm collections. Throughout the mid- to late 1900s, germplasm collecting efforts were prioritized for breeding programs to archive precious material before it disappeared and led to the development of the numerous large germplasm resources now available in different countries. In recent decades, however, the maintenance and particularly the expansion of these germplasm resources have come under threat, and there has been a significant decline in investment in further collecting expeditions, an increase in global biosecurity restrictions, and restrictions placed on the open exchange of some commercial germplasm between breeders. The large size of most genebank collections, as well as constraints surrounding the availability and reliability of accurate germplasm passport data and physical or genetic characterization of the accessions in collections, limits germplasm utilization by plant breeders. To overcome these constraints, core collections, defined as a representative subset of the total germplasm collection, have gained popularity. Core collections aim to increase germplasm utilization by containing highly characterized germplasm that attempts to capture the majority of the variation in a whole collection. With the recent availability of many new genetic tools, the potential to unlock the value of these resources can now be realized. The Commonwealth Scientific and Industrial Research Organisation (CSIRO) cotton breeding program supplies 100% of the cotton cultivars grown in Australia. The program is reliant on the use of plant genetic resources for the development of improved cotton varieties to address emerging challenges in pest and disease resistance as well as the global changes occurring in the climate. Currently, the CSIRO germplasm collection is actively maintained but underutilized by plant breeders. This review presents an overview of the Australian cotton germplasm resources and discusses the appropriateness of a core collection for cotton breeding programs.

## Introduction

By 2050, the world population is estimated to reach 9.1 billion people, and an increase of 70% in agricultural production is needed to feed and clothe the world (FAO, 2009; Tester and Langridge, 2010; Llewellyn, 2018). Land and water availability, climate change, and evolving pest and disease virulence are significant factors that add pressure to already intensive agricultural production systems. Plant breeding activities will be a critical avenue in addressing these challenges. The selection of traits for future climates such as abiotic and biotic stress tolerance, water use efficiency, and adaptation to new farming environments and systems could develop climate-resilient plant varieties. Target traits could be introduced from the naturally occurring diversity within a crop species, by utilizing the secondary and tertiary gene pools (Maxted et al., 2016) or through induced variation (Holme et al., 2019).

Plant genetic resources (PGR) constitute the foundation of sustainable agriculture and global food security and stability (Bernard et al., 2020). While the need for conservation and use is apparent, the lack of funding is severely impacting conservation efforts across the globe (Anđelković et al., 2020). Inadequate evaluation and characterization of germplasm held in genebanks are key challenges contributing to the underutilization of material and negatively impact future opportunities and funding efforts (Bernard et al., 2020). PGRs are the link between agriculture, environment and trade, so conservation efforts require cooperation from different sectors (Anđelković et al., 2020).

Since the 1970s, the conservation of PGRs has become a large-scale independent activity detached from crop improvement efforts. The germplasm held in genebanks is utilized by plant breeding organizations, often globally, and breeding programs are reliant on the genetic diversity that is available (Rajasekharan, 2015). Elite crop cultivars are generally bred from a narrow genetic base and targeted to high-input-intensive agricultural production, decreasing their ability to adapt to changes in the environment. Efforts need to be increased and directed to utilize germplasm from the genebanks for future climate-resilient breeding targets.

Globally, approximately 7.4 million accessions are preserved in over 1750 genebanks (Upahyaya, 2015), with fiber crops constituting ~104,000 accessions in the collection (FAO, 2010). Fiber crops hold importance in terms of raw materials for the textile industry as well as other industries including building materials, cosmetics, medicine, and biopolymers. Total fiber production is predicted to increase from 50 million tonnes/year in 1999 to 130 million tonnes/year by 2050 (European Commission, 2015). Fiber crops are dominated by cotton (European Commission, 2015), and Brazil, China, India, Russia, the United States of America (USA), and Uzbekistan are the countries that contribute the greatest to cotton production. Although Australia only produces around 3% of the world’s cotton, it is a significant player in the global trade of cotton, usually ranking as the third or fourth largest exporter (Khan et al., 2020; Cotton Australia, 2022). Australian cultivars are generally superior to those from many other countries in both seed and fiber qualities (Kilby et al., 2013; Gapare et al., 2017). Approximately, 1% of the global cotton accessions are held in Australian germplasm stocks (Campbell et al., 2010; Abdurakhmonov, 2014).

Cultivated cotton encompasses four species from the cotton genus, *Gossypium*; *G. arboreum* L. (Desi cotton)*, G. herbaceum* L. (Levant or Arabian cotton)*, G. barbadense* L. (Pima, Egyptian or Sea Island cotton), and *G. hirsutum* L. (Upland cotton; Wendel et al., 2009; Constable et al., 2015). Globally, *G. hirsutum* is the dominant species used in cotton production systems, making up around 95% of global cotton production (May and Lege, 1999; Zhang et al., 2008). However, the varieties released represent only a small portion of the total variation in the cotton gene pool (Wendel et al., 1992; Shim et al., 2018). This is largely due to the negative effects of linkage drag, deleterious alleles, and the breakdown of co-adapted gene complexes that can be introduced from wild germplasm (Acquaah, 2009) and breeders on the whole shy away from incorporating very unadapted germplasm into their breeding programs except in dire need.

Cotton breeding activities around the world have developed varieties with adaptation to new environments and modern management systems. Despite the past success, the full potential of future Australian varieties is constrained by imminent adverse climates influenced by climate change, changing biotic and abiotic stresses, and legislation and commercial developments that constrains resources used in cotton production (e.g., water, and fertilizers). The continued development of productive cultivars will rely heavily on exploiting the available variation present in conserved *Gossypium* species germplasm.

This paper aims to build on previous reviews on the purpose and relative benefits and challenges of germplasm collections (Fu, 2017; Díez et al., 2018; Walters et al., 2018; Wambugu et al., 2018; Westengen et al., 2018; Gotor et al., 2019; Hanson and Ellis, 2020). The outcome of the review will allow researchers to make an informed decision on the applicability of core collections for their situation. Such informed decisions are vital for plant breeding efforts, and germplasm collections as an effective core collection can provide a workable solution that ensures trait diversity can be harnessed in cultivar development programs.

## Core Collections

A plant germplasm collection is defined as a collection of seeds or breeding material, typically to conserve genetic diversity, but to also act as a source of material to address future challenges in agriculture (van Hintum et al., 2000). Germplasm banks generally hold a wide range of targeted cultivated species and wild relatives. Globally, the key germplasm resources for cotton are based in China (Jia et al., 2014), India (Narayanan et al., 2014), Pakistan (Rahmat et al., 2014), France (Dessauw et al., 2004), Uzbekistan (Sanamyan et al., 2014), Australia (Stiller and Wilson, 2014), and the United States (Percy et al., 2014; Zeng, 2014).

Although genebanks around the world have had great successes in collecting germplasm to conserve crop genetic diversity, the scale of success is often limited by inefficient data and germplasm management. The germplasm collections often become so large that maintenance of the conserved germplasm becomes difficult both from lack of resources and accumulated human errors in processing and handling, and potential contamination during seed regeneration cycles (Chen et al., 2016). In addition, when the data associated with germplasm lacks detail and the germplasm is under-characterized and under-evaluated, the germplasm is generally underutilized by breeders (Egan et al., 2019a,b, 2020). The ever-increasing gain that is needed from modern cultivars across all major crops suggests that the genetic variation available through germplasm banks will be even more critical for future breeding activities.

One method to tackle the management of large germplasm collections was suggested by Frankel (1984), through the development of a core collection. It was proposed that a pruned down collection would increase the management efficiency of the accessions and be more attractive to breeders. A core collection is defined as, “a limited set of accessions representing, with a minimum of repetitiveness, the genetic diversity of a crop species and its wild relatives.” The accessions that did not make it into the collection would not be discarded but would be managed in a “reserve collection.” The accessions chosen for the core collection should be genetically, ecologically, and geographically distinct from each other to maximize the diversity represented in the collection (Brown, 1995).

From the definition by Frankel (1984), several operational definitions have followed. For an individual genebank, van Hintum et al. (2000) proposed a core collection “consists of a limited number of accessions from an existing collection, chosen to represent the genetic spectrum in the whole collection”; this is termed the core subset of the collection, or is commonly referred to as a diversity panel. In comparison, a species-specific collection is made up of entries chosen to represent the majority of the genetic diversity of the target species and its wild relatives. It is a comprehensive collection and is often multi-organizational and international. Core collections now exist in chickpea, mungbean (Schafleitner et al., 2015), peanut (Holbrook et al., 1993; Jiang et al., 2014), apple (Liang et al., 2015), safflower (Dwivedi et al., 2005), cowpea (Mahalakshmi et al., 2007), pearl millet (Bhattacharjee et al., 2007), Australian bermudagrass (Jewell et al., 2012), common bean (Kuzay et al., 2020), annual Medicago (Diwan et al., 1994), capsicum (Zewdie et al., 2004), and eggplant (Miyatake et al., 2019). The most notable core collection is the International Barley Core Collection of some 1,600 accessions from around the world (van Hintum et al., 2000).

The development of “subset collections” and mini-core collections has also increased in recent years in response to germplasm management resource limitations (Pande et al., 2006; Sharma et al., 2010, 2012a,b). A subset collection is defined as a core collection for a specific breeding target and would be appropriate for an emergency situation, i.e., a disease decimates a crop, and a source of resistance is urgently needed to initiate a new breeding activity (Mahuku et al., 2003; Silvar et al., 2010). The concept of a mini-core collection is often attractive as the core collections are frequently still too large for the available resources to deeply characterize all of the accessions. Whilst a core collection is generally in the order of 10% of the size of the whole germplasm collection, a mini-core collection aims to subsample the core collection to develop a smaller collection (10% of a core collection) that captures most of the beneficial variation within the crop (Sharma et al., 2012a; Upadhyaya et al., 2013). Mini-core collections have been developed for chickpea (Upadhyaya and Ortiz, 2001; Pande et al., 2006), sorghum (Sharma et al., 2010), rice (Li et al., 2010), durum wheat (Etminan et al., 2017), mungbean (Sokolkova et al., 2020), cowpea (Fatokun et al., 2018), flax (Diederichsen et al., 2013), and finger millet (Upadhyaya et al., 2010).

In cultivated cotton, there are currently few core collections that exist globally (Xu et al., 2006; Mei et al., 2012; Wang et al., 2013; Ma et al., 2018). However, in Australia, there are as yet no core or mini-core collections for cotton.

## Current Germplasm Utilization Within Australian Cotton Germplasm Collections

Historically, Australian plant breeders across a range of crops acquired and maintained their own germplasm collections. However, in the early 1980s, the Commonwealth and State governments established a series of genetic resource centers to conserve national germplasm collections. The Australian Tropical Crops and Forages Genetic Resource Centre (ATCF) based in Queensland was the center that included cotton, although that collection has now been transferred to the Australian Grains Genebank in Horsham, Victoria, as part of a national consolidation effort. Currently, the only other two cotton-focused collections in Australia reside at the Commonwealth Scientific and Industrial Research Organisation (CSIRO) Agriculture and Food in Narrabri, New South Wales and Canberra, Australian Capital Territory.

The CSIRO main germplasm collection at Narrabri is maintained and funded by the active industry breeding program and includes about 2000 accessions. The CSIRO collection in Canberra is largely dedicated to the long-term cold storage of indigenous Australian species that have been collected through germplasm explorations over the last few decades, although nearly all are also entered in the US National Cotton Germplasm Collection. The majority of accessions held in Narrabri are cultivated tetraploid cottons, predominantly cultivars and germplasm stocks of *G. hirsutum* and some *G. barbadense*, with a small number of undomesticated or landrace stocks. The next largest group of accessions belong to the cultivated diploid cottons, with the majority being *G. arboreum* and a smaller number of *G. herbaceum*. A significant number of accessions of the Australian species are also held, particularly *G. australe* L., *G. sturtianum* L., *G. nelsonii* L., and *G. bickii* L. However, these are the result of individual germplasm explorations, so it is unknown what genetic variability exists between the accessions. Small numbers of accessions of most of the other *Gossypium* species are also held (Stiller and Wilson, 2014).

The majority of the successes in utilizing germplasm resources globally have been through the identification of important traits within the cultivated tetraploid species, the primary germplasm pool. From a breeding perspective, it is relatively straightforward to transfer traits from any of the main tetraploid species to *G. hirsutum*, the target of most commercial cotton breeding programs. Significant improvements in fiber quality have been realized through the introgression of traits from *G. barbadense* into *G. hirsutum* (Niles and Feaster, 1984; Meredith, 1991). In Australia, the most significant successes have been around improvements in disease resistance, particularly against Bacterial Blight, Fusarium Wilt, Verticillium Wilt, and Cotton Bunchy Top, mostly derived from other *G. hirsutum* cultivars.

The cultivated diploid cotton species, the secondary germplasm pool, are also of interest to cotton breeders as a source of novel traits. However, the challenges associated with ploidy differences make successful utilization difficult and a long-term exercise (often more than 20 years). Over the previous few decades, there have been numerous reports of attempts to transfer useful traits from these species (Stewart and Hsu, 1978; Brubaker et al., 1999; Liu et al., 2015a), but documented successes in terms of commercial cultivar releases are limited. However, one prominent example is the introgression of genes from *G. arboreum* and *G. thurberi* into Upland cotton to improve fiber strength (Culp and Harrell, 1974). In more recent times, the focus has shifted to pest and disease resistance and water and heat tolerance traits from the secondary gene pool (Constable, 1998; Singh et al., 2007; Azhar et al., 2009; Cottee et al., 2010, 2014; Trapero et al., 2016; Li et al., 2020; Wilson et al., 2021). This is also relevant to the Australian context where significant resources have been focused on using diploid species to improve resistance to Fusarium wilt and Verticillium wilt, as well as pests such as mites and whitefly.

The tertiary gene pool, including the many indigenous Australian *Gossypium* species, has also held great interest. The characterization of these diploid species has been reasonably well documented (Brown and Brubaker, 2000; Brubaker and Brown, 2003; Becerra Lopez-Lavalle et al., 2011) along with techniques for the development of fertile triple hybrids (Brubaker et al., 1999). Despite extensive research, there are no documented cases of the successful development of a cultivar utilizing these tertiary gene pool resources mainly due to the lack of any recombination of the diploid derived chromosomes with those of tetraploid cotton. However, considerable interest in this area remains and the continued development of advanced breeding techniques such as gene editing may eventually facilitate success in this area.

Historically, genuine germplasm exchange was practiced widely across breeding programs to support genetic gain on a global scale. However, cultivar development in many major field crops, especially corn, soybean, and cotton, is now heavily dominated by multinational corporations who generally adopt the approach of restricting access to their germplasm through various intellectual property protection strategies such as patents and exclusive licensing arrangements. This approach is understandable and has the potential to raise investment in cultivar development as realized economic returns can be reinvested back into research. The downside is an overall reduction in the movement of germplasm between programs and geographies, resulting in some genetic gains not being available to the global breeding community. As many cotton cultivars are only released with genetically modified traits, the germplasm exchange procedure is further complicated. The restricted movement of germplasm may also result in an overall slowing of genetic gains over time if individual programs become “isolated” and their overall genetic variability is reduced. Thus, it is an imperative that germplasm exchange continues. While there is no simple solution to this, one approach is for breeding programs to partner with these corporate programs or license in germplasm from other programs.

Another potential impediment to the utilization and expansion of germplasm collections as well as germplasm exchange between institutions is the Nagoya Protocol, part of the United Nations Convention on Biological Diversity (CBD). The Protocol is a multilateral international treaty with the purpose of ensuring “fair and equitable sharing of benefits arising out of the utilization of genetic resources,” which covers all organisms, including wild accessions of cotton and its relatives, wherever they are found. Researchers collecting new germplasm from the wild will have to enter into “access and benefit sharing” arrangements with the host country prior to their collection expeditions. These arrangements set out who might profit, and how, from the organisms being used, and stipulate how to distribute the benefits fairly, for example, through co-authorship of publications, or more importantly sharing of profits from products such as newly bred crops or new medicines. Although Australia signed and ratified the CBD in 1993, it is not a signatory to the Nagoya Protocol which was added in 2010 as it already had existing National and State and Territory laws consistent with the Protocol that regulate benefit sharing from any new collections of Australian indigenous biota, including *Gossypium* species. There is still some uncertainty around the interpretation of the Nagoya Protocol in relation to historical collections assembled from before the CBD (Sherman and Henry, 2020), so going forward some care will be needed by breeders in using wild germplasm in breeding, regardless of its origins, unless they can demonstrate Nagoya compliance or at least compliance with local national laws on access and benefit sharing.

## The Development of a Core Collection

**Figure 1:**
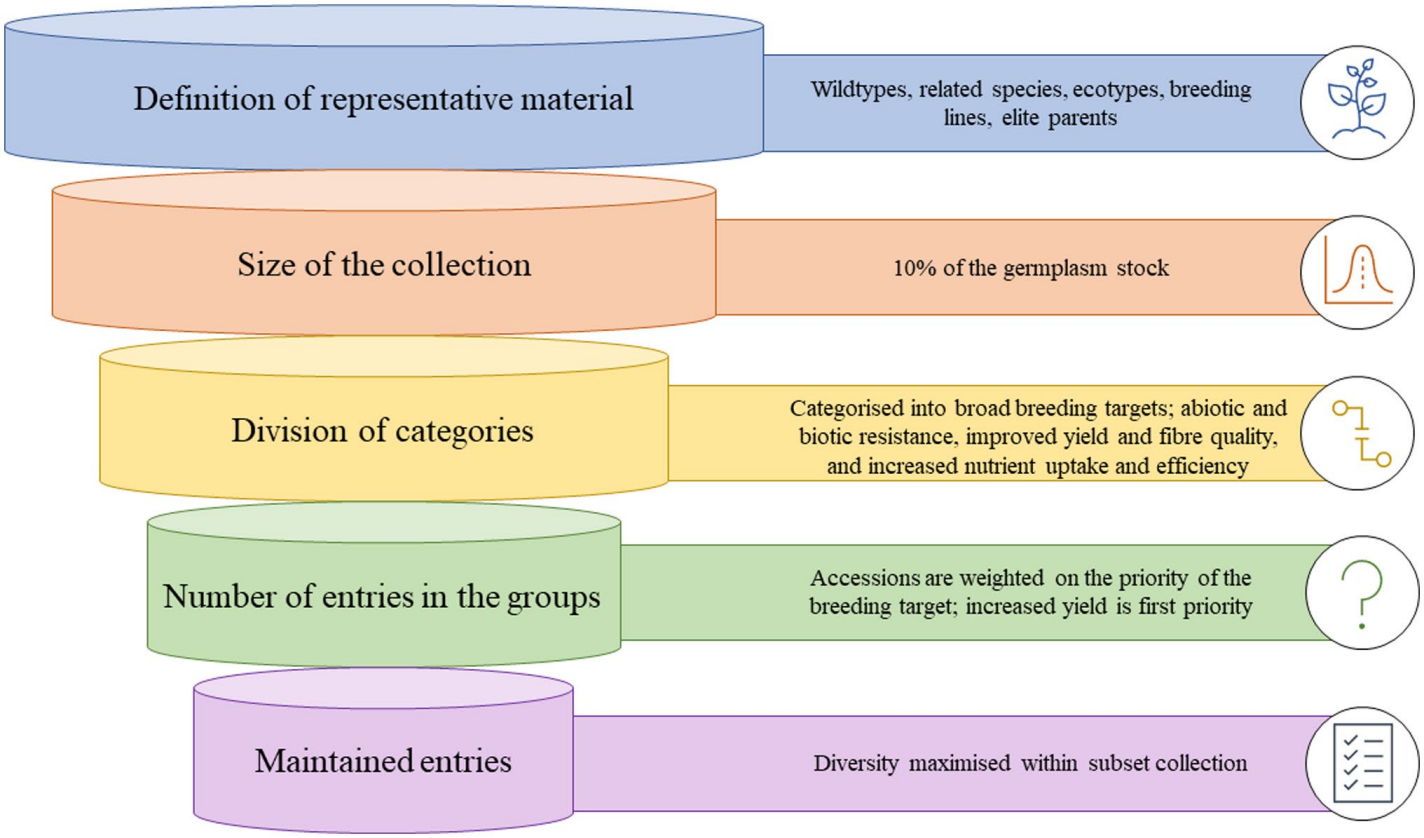
Graphic representation of how a core collection of cotton germplasm could be developed from entire germplasm stocks.

Since the proposal of a core collection, there have been many techniques and sampling methodologies developed. Creating a core collection can be simple or complex and is heavily influenced by resource allocation and information available. Therefore, often a preliminary core collection is developed, that is larger than intended, and then characterized at a deeper level and subset. In general, five generic steps are followed (Figure 1; van Hintum et al., 2000).

**Step 1**: *Definition of the material that should be represented*; this is termed the domain of the core collection and can range from related species, wild-types, ecotypes, landraces, previous breeding lines or a specific focus area of the diversity of a species. The definition is subjective to the available material, what constitutes a suitable set of material for core development, and the objectives behind the establishment of a core collection.

**Step 2:** *Decide on the size of the core collection*. Generally, most core collections are 5–20% of the size of the collection from which they were derived, although can be much smaller if the main collection is very large. A sampling strategy should define both a sampling method and an allocation method, and various sampling strategies and methods have been utilized to develop core collections (Franco-Duran et al., 2019). Based on the neutral allele model, a core collection of 10% of the total collection is likely to contain at least 70% of the variation of the collection, with an ideal collection size of no more than 3,000 accessions (Brown, 1989a,b).

**Step 3:** *Division of the domain into categories*. This is a methodical process and begins by dividing the material based on broad themes and further characterizing the groups. At every division, the groups should be constructed to maximize variation between the groups.

**Step 4:** *Decide on the number of entries in the groups of the core collection.* The number of entries of the core collection is heavily influenced by the aim of the core collection, i.e., the breeding targets. The allocation of germplasm across the defined groups is critical to maintaining diversity, and three generic steps have been proposed to determine the number of entries per group: (1) allocate entries in some proportion according to the relative numbers of accessions that occur within each group; (2) if enough material has been characterized with genetic markers, comparisons of marker diversity within the groups and basing the allocation of numbers based on maximizing allelic diversity or richness within each group; (3) subjectively consider the breeders needs and other informal knowledge about the accessions within the groups, i.e., passport data, availability to the collection curator.

**Step 5:** *Choose the specific entries that will be maintained in the core*. The final process of choosing which actual accessions should be included in the groups, maximize diversity, and serve the purpose of the core collection. However, breeders also need to consider the quality of the documentation of the entries, seed availability, and the role of the accessions in their breeding programs (van Hintum et al., 2000).

## Cotton Core Collections as a Resource to Discover New Sources of Variation for Biotic and Abiotic Stress Tolerance

Plants are sessile organisms and thus are exposed to environmental conditions and biotic stressors that are detrimental to their growth and development. Improving germplasm to meet abiotic and biotic breeding targets is a long-term process and can involve extended time and resources for successful introgression into elite material. However, there are many examples where plant breeders have effectively utilized well-characterized germplasm from a germplasm center or core collection to introgress tolerance/resistance to abiotic and biotic stresses in crop species (Upadhyaya et al., 2011; Prasanna, 2012; Shivhare and Lata, 2017; Singh et al., 2018; van Zonneveld et al., 2020; Sharma et al., 2021). Climate-proofing future cotton varieties will heavily involve the incorporation of traits and sources of resistance to biotic and abiotic stresses.

### Biotic Resistance

One of the most important roles for a core collection is when a new disease, or novel variant, threatens the industry and necessitates the rapid establishment of a breeding project to confer host plant resistance. However, biotic stresses are complex due to the high interaction of the pest or disease with the plant, the environment, and evolving pest and disease virulence. Currently, the diseases of Fusarium wilt, Black Root Rot, and Verticillium wilt, and the increase in secondary pests with the introduction of genetically modified cotton resistant to the major Lepidopteran pests are the greatest biotic threats to the Australian cotton industry. A good example of the utilization of germplasm to address a rising disease threat is our breeding for Fusarium wilt resistance in cotton.

Fusarium wilt is caused by the pathogen *Fusarium oxysporum* f sp. *vasinfectum* and was first recognized in Australia in 1993 (Kochman, 1995) when most of the commercially grown cultivars were highly susceptible. Production losses of up to 100% were reported from fields with high levels of inoculum. It is thought that the indigenous and genetically unique Australian Fusarium wilt (Wang et al., 2004) pathogen became prominent in response to wide-scale planting of highly susceptible cotton cultivars and then spread to nearly all cotton-growing regions *via* infected soil, flood waters, or on cotton trash. An initial screen from the main CSIRO germplasm collection of *G. hirsutum* genotypes from the United States, South America, Africa, Asia, and Europe revealed that most were susceptible, but a few genotypes showed field survival up to 50% higher than the best extant Australian cultivars. Over the next years, over 200 genotypes from the collection were targeted for evaluation based on material from the countries/regions/programs that showed promise in the early screening. One line from India MCU-5 (Lopez-Lavalle et al., 2012) possessed significantly higher levels of resistance and became the initial source of resistance in a breeding process with moderately resistant local elite varieties. The introgression of MCU-5 germplasm ultimately resulted in the release of Sicot F-1 in 2004, which had at least twice the resistance to Fusarium wilt of the best cultivar in 1994 (Allen et al., 2004).

Screening our germplasm collection has also identified genotypes for breeding purposes with increased resistance to Cotton Bunchy Top disease (Ellis et al., 2016) and Bacterial Blight (Rungis et al., 2002), and more recently Verticillium wilt.

### Abiotic Resistance

The major abiotic stressors affecting the Australian cotton industry include temperature extremes, water availability, salinity/sodicity, mineral toxicity, and UV radiation. Ultimately all abiotic stressors adversely alter morphological, biochemical, and physiological plant mechanisms, resulting in reduced plant growth and productivity, preventing crops from reaching their genetic yield potential. Water stress is the major environmental factor influencing crop productivity, where approximately 40% of the global land area is affected by drought (Trenberth et al., 2014). Thus, significant research has been dedicated to the development of drought-tolerant cotton germplasm and due to its complexity is an interesting case study for the development of core collections.

Like most core collections, passport data (such as the climate of origin and/or target environments of breeding lines) provide a good basis for the initial development of a drought-tolerant cotton mini-core collection. In addition, landrace accessions and cotton wild relatives are essential for the inclusion as they may have drought traits that have been lost from cultivated varieties due to selection pressure for yield potential under optimal conditions (Dempewolf et al., 2017). The yield performance of germplasm under controlled field conditions provides additional confidence in the development of a drought-focused core collection. Under water-limited conditions, the genetic potential of a genotype may not be expressed, which limits the ability to resolve statistical genotype differences. This is particularly important in variable rainfall environments and can be mitigated through the implementation of a managed stress environment (MSE) protocol. An MSE protocol ensures water is applied to “rainfed” germplasm trials when crop yield is likely to fall below the threshold for resolving genetic differences with confidence (Conaty et al., 2018). An MSE protocol has been used to identify germplasm for our drought tolerance research using a germplasm panel that could be utilized as a mini-core collection. In addition, germplasm evaluations are conducted in multiple rainfed and irrigated environments, allowing for paired comparisons and the development of drought resistance indices used in selection, e.g., Fischer and Maurer (1978) and Mwadzingeni et al. (2017).

Several institutions have developed core collections in other crop species with increased tolerance to abiotic stresses: chickpea (Upadhyaya et al., 2013), sorghum (Upadhyaya et al., 2009), barley (Muñoz-Amatriaín et al., 2014), bermudagrass (Anderson, 2005), and peanut (Upadhyaya et al., 2014). These activities have had promising results and should encourage similar efforts in cotton.

### Variation in the Cotton Genome

Before the advent of molecular genetics, plants were assessed on their diversity in morphological and phenotypic traits as well as their pedigree and geographical distribution. Viewed molecularly, cotton diversity is the variation of the genes within each species. Recent advances in technology have resulted in ever-decreasing costs of high-throughput sequencing that has enabled the assembly of allopolyploid cotton reference genome sequences in a restricted set of “standard” genetic lines (Li et al., 2015; Liu et al., 2015b). These standard lines provide only a snapshot of cotton genome structure and so it is only recently with the rise of third-generation sequencing (long-read sequencing), that the true diversity within each species can be viewed through the completion of pan-genome sequences (Bayer et al., 2020). Pan-genomes represent the genomic diversity of a species that includes core genes, found in all individuals, as well as variable genes, which are present–absent in some individuals and are likely the result of local adaptations. A simple comparison of two *G. hirsutum* cultivars TM-1 (the genetic standard for *G. hirsutum*) and zhongmiansuo24, a commercial cultivar (Yang et al., 2019) revealed extensive gene order and gene structural diversity, and in more extensive comparisons in other crop species, there has been large variable gene content (15–40% gene presence and absence variation) between individuals. Although pan-genomes are still in their infancy for many crop species, a pan-genome approach should enable an informed choice for the selection of core collection accessions based on both gene sequence and gene presence/absence diversity between individuals or accessions. For disease-resistant plants, for example, NOD-like receptor (including disease resistance like genes) pan-genome diversity (Barragan and Weigel, 2020) could be used to prioritize lines for a core collection. Molecular surveys of diversity, therefore, could enable the selection of core lines that encompass species diversity without the requirement for extensive phenotype analysis.

## The Challenges of Developing a Core Collection

Although core collections have the potential to address the underutilization of germplasm that is a frequent challenge for genebanks, there are concerns from breeders about certain aspects of the development stage. Three key areas highlighted by Brown and Spillane (1999) are the evaluation criteria used, completeness of data, and the disposal of germplasm not in the core collection. First, the choice of traits to be incorporated into the core collection needs to be flexible. Many breeders approach genebank curators with specific traits in mind that they want to incorporate into their program, and if the core collection does not contain the desired traits then germplasm utilization will remain stagnant (Egan et al., 2019a). The collection also needs to have the ability to pivot direction if new breeding targets and trends emerge (Brown and Spillane, 1999). Second, the core collection may lack crucial accessions if it was formed using incomplete information (Brown, 1995). If the collection was developed using misleading or incomplete information, then it could lead to a false assumption that the core collection is a broader sample than it actually is Brown and Spillane (1999). Finally, there are concerns about the potential disposal of accessions that are not included in the core. The intention of a core collection is not to replace the main collection but to increase its utilization, so curators should make every effort to maintain underutilized accessions in longer-term storage as part of the main collection, perhaps with more restricted access by users, or to transfer them to other institutions with greater resources rather than dispose of any accessions due to resource constraints (Brown, 1989b; Brown and Spillane, 1999). Therefore, there is a stronger case to develop a core collection to utilize some of the germplasm rather than have none of it used at all. Overall, when a germplasm center decides to develop a core collection, the risk to the whole collection is low, at least for the major crops (Hamon et al., 1995).

While the concept of identifying germplasm that is resistant to abiotic or biotic stressors to include in the core collection is hopeful, there are significant challenges associated with identifying said germplasm. The two major challenges are: (1) identifying a source of resistance to the stress and (2) developing an effective screening method to characterize the level of resistance. The development of a core collection for resistance to an abiotic or biotic stress is limited by the variation available in germplasm. In some circumstances, particularly for biotic stresses, there may not be any resistant germplasm in either the main or core collection and the pest or pathogen will be better controlled by management strategies. Therefore, a wide screen of the germplasm available using an appropriate phenotyping method is critical before deciding if a core collection is an effective solution. Once a source of resistance is identified, a high-throughput screening assay that is reflective of field resistance needs to be developed. However, this is often a lengthy process and is complicated by several factors. First, the susceptibility of plants to the stress varies with respect to timing, duration, and intensity and often under field conditions, plants encounter specific abiotic and biotic stresses in combination with other stressors. Second, controlled environment and pot-based experiments are generally not well correlated with field performance. Finally, resistance to abiotic and biotic stressors is often polygenic, including a complex of gene networks involved in stress sensing, signal transduction, and expression of stress-responsive genes. Therefore, the development of core collections to assess a wide range of germplasm rapidly and effectively for abiotic and biotic stress resistance can be an ambitious goal.

While core collections for drought tolerance have been assembled, gains in limited water and rainfed cotton productivity have largely been incremental and reflective of improvements in yield potential under non-stressed conditions. Thus, future genetic gain in abiotic stress resistance will require a combination of traditional plant breeding and new breeding methods such as genomic selection, as well as the integration of panomics (Weckwerth et al., 2020), novel field-based phenomics (White et al., 2012; Zhao et al., 2019), and the application of machine learning and artificial intelligence to breeding for complex plant traits (Niazian and Niedbała, 2020; Nabwire et al., 2021). However, these methods are currently prohibitively expensive and/or will require a core collection of diverse germplasm to efficiently assess whether they will be effective in cotton breeding.

The major bottleneck for the development of core collections remains the required level of investment. Developing and maintaining a germplasm collection is time-consuming and expensive, and there is a clear reluctance to investment due to the large cost of germplasm maintenance and the continual characterization of germplasm for numerous traits. Adding to the expense is the high likelihood that novel resistance traits will often lie in related cotton species, rather than older varieties or landraces, and characterizing and introgressing resistance from those more distant sources is a lengthy and costly process. To encourage funds to be allocated to germplasm centers, the development of a core collection must show a clear return on investment and have an applied and impactful outcome to commercial breeding programs.

## Will Core Collections Provide Benefit to Australian Cotton Breeding Activities?

The challenges facing the Australian cotton industry include increasing production costs, pests, and diseases, and new environmental conditions due to climate change. While these challenges are both numerous and diverse, a core collection would only be useful to the CSIRO breeding program if it was a single collection that contained deeply characterized accessions for multiple traits. This is because the fundamental focus of the breeding program is the production of commercial cotton varieties, not germplasm banking. Thus, the investment required for the core collection must match the desired outcome and provide impact in terms of cultivar development. However, it should be acknowledged that the approach used for trait screening and ultimately cultivar development over the last few decades by CSIRO has utilized the central principles involved in developing a core collection. The screening and characterization of approximately 200 diverse accessions for Fusarium wilt resistance (see “Biotic Resistance,” above) are a good example of how the principles of developing a core collection can be employed in breeding.

Although yield will always be the main priority for the CSIRO cotton breeding program, the core collection would not directly target high-yielding accessions. Rather, the traits targeted would include those with less complex genetic backgrounds, such as biotic stress resistance and the fiber quality parameters of length, strength, and elongation. It is an ambitious goal that abiotic stress tolerance would be included as a characterization parameter due to the complexities in phenotyping for that type of tolerance. It is probable that the categories across the core collection would contain an unbalanced number of accessions as target traits may only be present in low frequencies throughout our entire germplasm collection. Conversely, for traits that are common, the highest performing accessions would be selected for inclusion in the core collection. Importantly, adequate representation from related cotton species must be included, historically sources of host plant resistance to biotic stressors have been identified in landrace and crop wild relatives in the CSIRO cotton breeding program (Allen et al., 2004; Miyazaki et al., 2012; Wilson et al., 2021). We conclude that CSIRO should pursue the development of a core collection through a greater characterization of our main collection at both a molecular and phenotypic level and augmenting it where necessary with new material from global collections to fill gaps and ensure a high level of genetic diversity across several traits types of interest, but of a size that we can economically maintain through regular regeneration of our stocks.

## Conclusion

Conserving germplasm is, in the long term, the most efficient and inexpensive method of genetic conservation for agriculturally important plants. However, the conservation of germplasm quickly becomes unmanageable, and accessions will be underutilized if a cost-effective management system is not implemented. Core collections are one strategy to increase the utilization of germplasm and have moved from a period of experimentation to one of increasing popularity and endorsement.

The development of core collections presents great opportunities for cotton and provides a resource use-efficient strategy enabling the identification of the most accessible source for a given trait (Brown, 1989b). Core collections that represent the majority of the diversity available in cotton would demonstrate significant value for germplasm conservation and breeding activities. Future-proofing the global cotton industry will rely heavily on the introgression of germplasm for novel traits, particularly focused on resistance to abiotic and biotic stresses.

Nevertheless, the significant challenges of developing a core collection must be clearly acknowledged. To be of real value, the core collection should target traits specific to that required by the industry that a breeding program is supporting. This will be different for different countries, regions, and production systems. Significant investment from the program would need to be allocated to develop and maintain the core collection. Therefore, the target traits must be a continuing important breeding goal to the relevant industry. Due to the vastly different cotton production systems across the globe, it is unlikely that a single global cotton core collection would provide merit to all breeding programs; rather, a country- and trait-specific core collection model would be more appropriate.

Finally, to fully exploit the potential that core collections can bring to a breeding program, coordinated efforts between the private and public sectors are required. The goal of these efforts is to ensure that meaningful germplasm access and exchange can occur, resulting in core collections that better represent the diversity of the focus traits of that specific core collection. However, it must be acknowledged that germplasm protection is fundamental for all commercial breeding programs. Therefore, access to the full range of germplasm is unlikely. This can in part be mitigated through commercial agreements providing access to recently released cultivars, and private sector breeding programs releasing commercially obsolete germplasm to publicly available germplasm libraries.

## Author Contributions

LE conceived and drafted the review. LE, WC, and WS designed and wrote the review. All authors contributed to the article and approved the submitted version.

## Funding

This work was supported by Cotton Breeding Australia, a joint venture between Cotton Seed Distributors Ltd. Australia and CSIRO Agriculture and Food.

## Conflict of Interest

The authors declare that the research was conducted in the absence of any commercial or financial relationships that could be construed as a potential conflict of interest.

## Publisher’s Note

All claims expressed in this article are solely those of the authors and do not necessarily represent those of their affiliated organizations, or those of the publisher, the editors and the reviewers. Any product that may be evaluated in this article, or claim that may be made by its manufacturer, is not guaranteed or endorsed by the publisher.

## Abbreviations

ATCF: Australian Tropical Crops and Forages Genetic Resource Centre
CSIRO: Commonwealth Scientific and Industrial Research Organisation
CBD: Convention on Biological Diversity
MSE: Managed Stress Environment
PGR: Plant Genetic Resources
USA: United States of America
